# A Glimpse into an Editorial Training Program: From the *Kidney360* Editorial Program Trainees

**DOI:** 10.34067/KID.0000000000000311

**Published:** 2023-11-20

**Authors:** Molly Fisher, Abinet M. Aklilu, Monica Suet Ying Ng, Denisha R. Spires, Miguel Bigotte Vieira

**Affiliations:** 1 Division of Nephrology, Albert Einstein College of Medicine, Montefiore Health System, Bronx, New York; 2 Section of Nephrology, Department of Internal Medicine, Yale School of Medicine, New Haven, Connecticut; 3 Kidney Health Service, Royal Brisbane and Women's Hospital, Herston, Queensland, Australia; 4 Conjoint Internal Medicine Laboratory, Chemical Pathology, Pathology Queensland, Herston, Queensland, Australia; 5 Faculty of Medicine, University of Queensland, Herston, Queensland, Australia; 6 Department of Physiology, Augusta University, Medical College of Georgia, Augusta, Georgia; 7 Nephrology Department, Hospital Curry Cabral, Centro Hospitalar Universitário de Lisboa Central, Lisboa, Portugal; 8 Nova Medical School, Lisboa, Portugal

**Keywords:** clinical nephrology

## Overview

*Kidney360* is the open-access journal of the American Society of Nephrology. Born on January 1, 2020, with the release of its first issue, *Kidney360* publishes content across all disciplines of kidney science. In the fall of 2021, *Kidney360* issued a call for applications for the inaugural Editorial Training Program (ETP) with the mission to equip early career nephrologists with necessary skills to become strong peer reviewers and editors. After receiving over 50 applications, three of us were selected to join a yearlong training program which commenced January 2022. After its initial year, the ETP increased in duration to 2 years, including four trainees with diverse backgrounds in general and transplant nephrology and clinical and basic science research. True to its name, *Kidney360* has made it possible for early career nephrologists from three continents of the world to take part in this rigorous training opportunity within 3 years of its inception. In this perspective, the trainees described our experience as part of the *Kidney360* editorial team, lessons learned, and advice for future trainees.

## Training at *Kidney360*

As ETP trainees, we are engaged at various levels of the editorial decision-making process from peer review to article triaging, reviewer selection, evaluation of the reviews, and final decision making with progressive increase in responsibility. Below, we highlight the five core components of the training program.

1. Peer review: The first year focuses on developing proficiency in peer review, a critical but rarely taught skill in academic medicine. Each trainee is assigned an Associate Editor (AE) who serves as their mentor. This starts with a joint review of 1–2 manuscripts per month to supplement reviews from outside experts. We review a broad range of submitted manuscripts across the clinical and basic science research domains, including original research, review articles, brief communications, and perspectives. We regularly meet with our mentor to discuss our assessment, compare our review to that of expert invited reviewers, and reach a final editorial decision. Biannually, we rotate mentors so that we are exposed to AEs with diverse areas of expertise within nephrology. This experience has taught us several important skills, including assessment of the scientific merit of an article and provision of efficient feedback to authors to help strengthen the presentation, content, and clarity of their manuscript.
2. Editorial selection: Editorials are an important way to communicate research findings. They serve to draw attention to new innovations and/or provide context to an evolving or controversial research topic. Every week, we review recently accepted *Kidney360* accepted manuscripts and provide a rationale for or against an Editorial or Podcast. Within a week, we receive feedback and decisions made by the Editor-in-Chief and Deputy Editors. Trainees also contribute to material selection for Perspective or Reviews in Editorial meetings with real-time feedback from Editors. This level of engagement with the editorial team keeps us informed on all upcoming publications and challenges us to think critically and creatively about the potential effects of recent discoveries.
3. Invited manuscripts: One of the most exciting aspects of the ETP has been the opportunity to present new ideas for invited review articles, perspective pieces, and debates. We present topic ideas and potential authors to the Editorial Team at monthly editorial teleconference meetings where we receive further feedback, including suggestions on authors. We get a chance to work with experts on the editorial board to develop these topics and invite authors with the assistance of the Editorial Manager. This provides us further opportunity to work with other AEs and learn about the process of identifying, selecting, and inviting authors with expertise in unique areas. This serves as an opportunity to become familiar with not only *Kidney360*'s but also other nephrology journals' contents and identify knowledge gaps and new research developments to advance kidney care. Article collections on xenotransplantation and women's health are some examples of ideas proposed by trainees that have now been published.
4. Editorial decisions: During the second year of training in the program, our responsibilities have expanded to mimic the role of an AE. Under the guidance of an AE, we perform initial review of select manuscripts, decide whether they should be sent for review or triaged, select suitable reviewers on the basis of the area of expertise, evaluate the completed reviews, and make the final editorial decision. This experience has given us an appreciation for the art of peer reviewer selection and reviewers who volunteer their time to evaluate the quality of a manuscript and its suitability for publication.
5. Social media: Our role at *Kidney360* continues to evolve, incorporating new projects to deliver and translate the *Kidney360* content for its readership. Motivated by the growing appreciation for social media in medicine, we launched *Kidney360* tweetorials in 2022 to facilitate knowledge translation and dissemination of recent publications. Our tweetorials simplify articles into educational threads of 10–12 tweets on various topics such as “*Fundamentals of Arterial Blood Gas Interpretation*” and “*The role of Peritoneal Dialysis in Different Phases of Kidney Transplantation*.” Trainees create the tweetorials and collaborate with a dedicated *Kidney360* social media team to publish them online. We also participate in the development of visual abstracts and podcast scripts for accepted articles. This has allowed us to develop skills in using social media platforms to disseminate scholarly work and effectively communicate key scientific findings, important skillsets in medical education and research.

**Figure 1:**
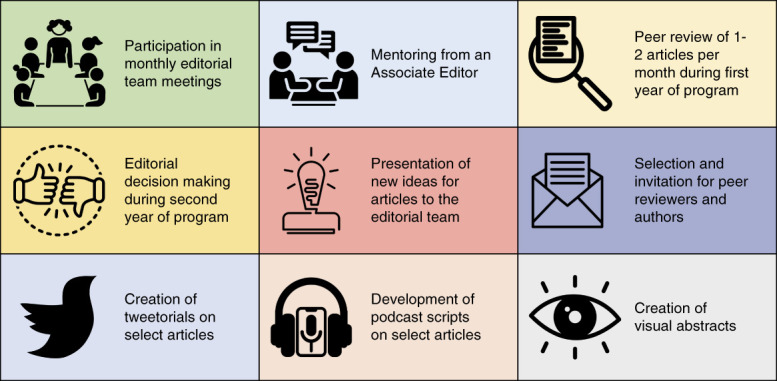
**Components of the *Kidney360* ETP.** ETP, editorial training program.

Figure 1 presents our roles in the program, which could serve as a blueprint for the design of other ETPs. The lessons learned are summarized in Box 1.

**Box 1. Lessons learned from core components of Editorial Training Program training**

**Table:** 

Core Editorial Training Program Component	Lessons Learned
Mentored peer review	• Assessment of the scientific merit of an article and provision of efficient feedback to authors to help strengthen the presentation, content, and clarity of their manuscript • Professional development • Mentorship skills
Manuscript selection for editorial accompaniment	• Keeps us informed on upcoming publications • Gain different perspectives • Challenges us to think critically and creatively about ○ Types of publications that warrant highlighting in Editorials or Podcasts ○ Topics that would be of interest to readers as a perspective or review
Social media	• Develop important communication skillsets in medical education and research ○ Disseminating scholarly work in an accessible manner ○ Effective communication of key scientific findings to a broader audience
Invited manuscripts	• Gain familiarity with *Kidney360's* and other nephrology journals' recent contents • Identify knowledge gaps and new research developments to advance kidney care • Learn about the process of identifying, selecting, and inviting authors with expertise in unique areas from multiple Associate Editors
Editorial decisions	• Develop an appreciation for the art of peer reviewer selection and to reviewers who volunteer their time • Skills in evaluating peer reviews and balancing diverging reviews • Develop skills in higher-level assessment of the quality of a manuscript and its suitability for publication

## Editorial Fellowships: Training the Next Generation

Many scientific journals offer editorial fellowships/ETPs to early career physicians. The *New England Journal of Medicine* started one of the first editorial fellowships in 2000 which paved the way for other medical journals.^1^ Editorial fellowships provide training on medical publishing, the path from research submission to publication, the appraisal and interpretation of manuscripts, and effective communication of research discoveries. From general medicine to subspecialty journals, these fellowships have grown in scope and in the number of participants. Other nephrology journals that offer editorial fellowships include *Kidney International*, *JASN*, *American Journal of Kidney Diseases*, and *Nephrology Dialysis Transplantation*.^2,3^ The growing number of editorial fellowships in nephrology will promote diversity and ensure the maintenance of high standards in research by training young physicians in sound research and communication, scientific writing, and mentorship.^4^

The *Kidney360* ETP, like any educational program, is shaped by each participant's involvement and dedication and the unwavering support of the editorial team who are committed to training early career nephrologists. Although the training is rigorous, the skillsets, including mentorship skills acquired along the way, will have long-lasting effect on future generations of clinicians and scientists. We strongly encourage future and early career nephrologists to seek out these training opportunities. Participation in an ETP provides an unparalleled advantage to learn firsthand the operations of a scientific journal and the editorial role. As the saying goes, “*We learn by doing*.” However, given the limitations in capacity barring many from participating in such programs, we include select resources,^5–9^ covering a range of topics, including conflicts of interest, authorship attribution, confidentiality, research and publication ethics, reducing bias in peer review, reading a paper, conducting a review, responding to authors, and appraising content integrity, accuracy, and completeness. We hope these will serve as a valuable resource to future editorial fellows and early career academic nephrologists. We also encourage other journals to provide this training opportunity to those interested in learning about the peer review process. Peer review is an important and rewarding responsibility to the medical community. We are grateful to the authors and peer reviewers who volunteer their time to help us ensure high-quality publications at *Kidney360*.

## Final Words from the Trainees

“It has been a great privilege to be part of *Kidney360* and to see our voices and ideas reflected in its content”—M. Fisher

“It has been an honor to be able to share my perspective as a young European nephrologist with the exceptional *Kidney360* Editorial Team”—M. Bigotte Vieira

“As an early career nephrologist training in clinical research, participating in the *Kidney360* ETP has been an incredibly rewarding learning experience.”—A. M. Aklilu

“The *Kidney360* ETP has provided invaluable insight into the facets of academic publishing through participation in a supportive learning environment”—M. Suet Ying Ng
